# Proresolving and cartilage-protective actions of resolvin D1 in inflammatory arthritis

**DOI:** 10.1172/jci.insight.85922

**Published:** 2016-04-21

**Authors:** Lucy V. Norling, Sarah E. Headland, Jesmond Dalli, Hildur H. Arnardottir, Oliver Haworth, Hefin R. Jones, Daniel Irimia, Charles N. Serhan, Mauro Perretti

**Affiliations:** 1 William Harvey Research Institute, Barts and the London School of Medicine, Queen Mary University of London, London, United Kingdom.; 2 Center for Experimental Therapeutics and Reperfusion Injury, Harvard Institutes of Medicine, Department of Anesthesiology, Perioperative and Pain Medicine, Brigham and Women’s Hospital (BWH) and Harvard Medical School, Boston, Massachusetts, USA.; 3 Center for Engineering in Medicine, Massachusetts General Hospital, Harvard Medical School, Shriners Hospital for Children, Boston, Massachusetts, USA.

## Abstract

Rheumatoid arthritis (RA) is a debilitating disease characterized by persistent accumulation of leukocytes within the articular cavity and synovial tissue. Metabololipidomic profiling of arthritic joints from omega-3 supplemented mice identified elevated levels of specialized proresolving lipid mediators (SPM) including resolvin D1 (RvD1). Profiling of human RA synovial fluid revealed physiological levels of RvD1, which — once applied to human neutrophils — attenuated chemotaxis. These results prompted analyses of the antiarthritic properties of RvD1 in a model of murine inflammatory arthritis. The stable epimer 17*R*-RvD1 (100 ng/day) significantly attenuated arthritis severity, cachexia, hind-paw edema, and paw leukocyte infiltration and shortened the remission interval. Metabololipidomic profiling in arthritic joints revealed 17*R*-RvD1 significantly reduced PGE_2_ biosynthesis, while increasing levels of protective SPM. Molecular analyses indicated that 17*R*-RvD1 enhanced expression of genes associated with cartilage matrix synthesis, and direct intraarticular treatment induced chondroprotection. Joint protective actions of 17*R*-RvD1 were abolished in RvD1 receptor–deficient mice termed *ALX/fpr2/3^–/–^*. These investigations open new therapeutic avenues for inflammatory joint diseases, providing mechanistic substance for the benefits of omega-3 supplementation in RA.

## Abstract 2

The lipid mediator resolvin D1 attenuates arthritis severity, limits joint leukocyte infiltration, and promotes cartilage protection.

## Introduction

Rheumatoid arthritis (RA) is a chronic debilitating disease characterized by persistent synovitis, which leads to joint damage, increased disability, and accelerated cardiovascular disease, leading to higher mortality (1). It is now appreciated that chronic inflammatory diseases such as arthritis may persist due to a failure of resolution responses (2). Current treatment strategies for RA aim to intervene early to consistently limit inflammation in order to maintain joint integrity (3). Accordingly, patient prognosis has dramatically improved since earlier diagnosis, along with the revolutionary advent of biologics and treatment with disease-modifying antirheumatics and methotrexate (4). Despite this, current expectations are that only 50% of treated patients will exhibit a reduction in disease activity score from high to a sustained level of low or, more rarely, remission (4). In addition, treatment controls yet seldom repairs joint damage (5), and patients on therapies with biologics develop an increased risk of opportunistic infections (4, 6). Thus, new therapeutic approaches that can counter disease chronicity and maintain joint integrity are required.

In order to circumvent the progress of acute self-resolving to persistent-chronic inflammation, the inflammatory response must be actively resolved. It is now appreciated that this process is not passive as once believed, but it is governed by the spatiotemporal production of endogenous proresolving mediators (2). Uncovering these mediators and elucidating their mechanisms of action has shed light on the promising nature of these compounds as potential therapeutics (7). Omega-3 polyunsaturated fatty acids (PUFA), which are abundant in marine oils, are precursors to a new genus of bioactive lipid mediators that comprise the lipoxins, resolvins (Rvs), protectins, and maresins (MaRs) (collectively termed specialized proresolving lipid mediators [SPM]; refs. 2, 7), which exhibit potent and stereoselective protective properties such as limiting further neutrophil recruitment, enhancing containment and clearance of infections, and reducing inflammatory pain (2, 7, 8). Dietary supplementation with fish oils has proved efficacious in reducing joint pain, swollen joint count and NSAID usage in RA patients, yet there is an unmet need to determine the mechanism of action(s) of these supplements (9, 10). Thus, SPM may offer a molecular mechanism and basis for the beneficial effects of omega-3 consumption in specific RA clinical endpoints (9, 10).

In the present study, we performed lipid mediator metabololipidomics on arthritic joints from mice fed standard versus omega-3–enriched chow to directly compare the local bioactive lipid mediator metabolome. Among the mediators quantified, we identified elevated levels of RvD1, which was also identified in human RA synovial fluids. When its 17*R* epimer (selected for its increased metabolic stability, ref. 11) was administered to mice during inflammatory arthritis, it significantly reduced joint inflammation. Herein, we report mechanisms of action of RvD1 and tested its antiarthritic potential in an inflammatory polyarthritis model evoked by transfer of arthritogenic serum that mimics the presentation and histopathology of human RA.

## Results

### Dietary supplementation with omega-3 PUFA reduces arthritis and modulates the local lipid mediator profile.

**Figure 1:**
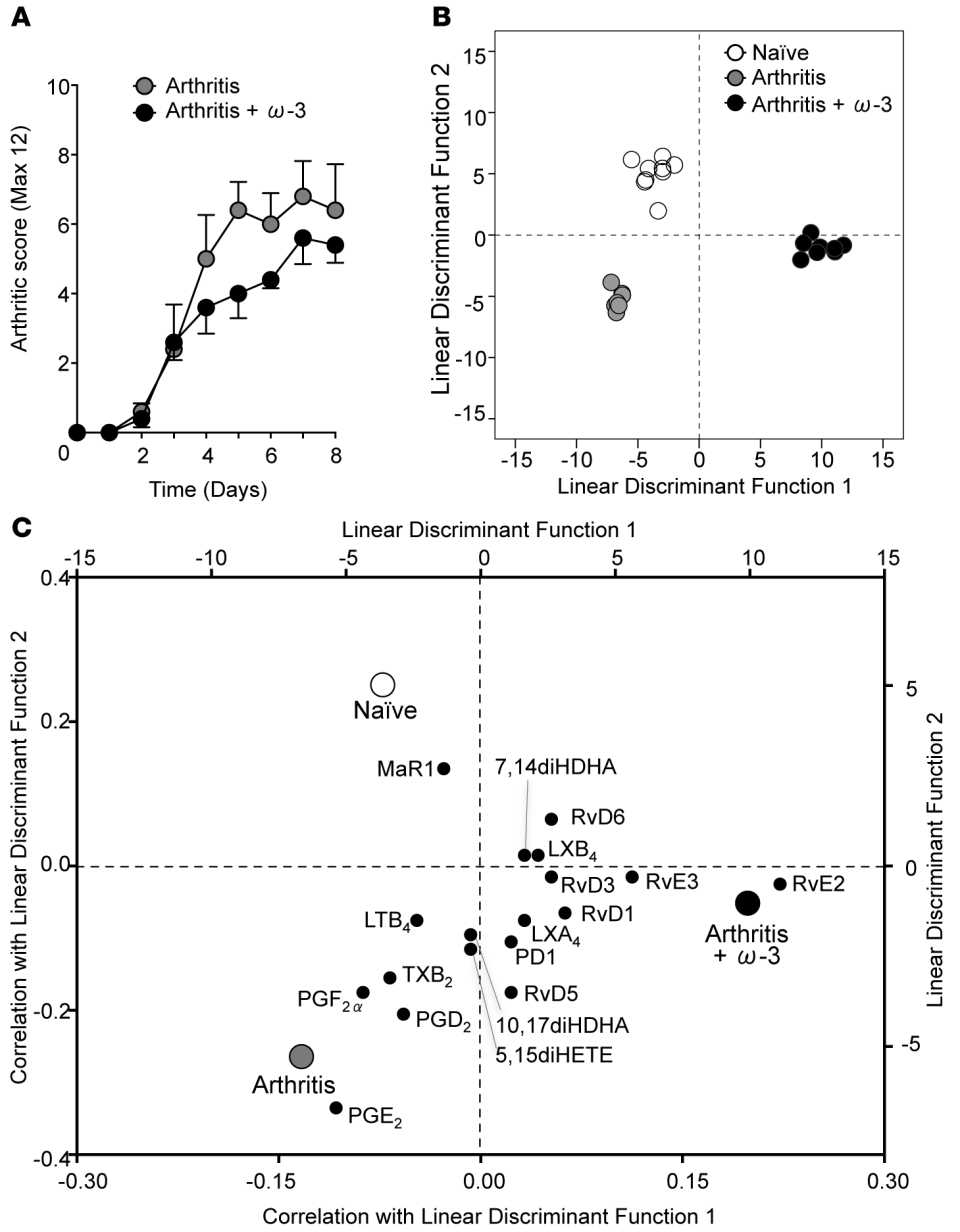
Mice fed an omega-3–supplemented diet display reduced arthritis and a modulated local biosynthesis of bioactive lipids within arthritic joints. Mice fed a standard or omega-3–supplemented diet were given arthritogenic serum (100 μl, i.p. on days 0 and 2) and (**A**) arthritic score was evaluated. (**B**) Arthritic paws were collected for metabololipidomics analysis on day 8. Linear discriminant analysis was used to generate 2 discriminant functions from the values of bioactive lipid mediators (as quantified by LC-MS/MS), which maximize the difference between naive, arthritis, and arthritis + omega-3 treatment groups. Each datum corresponds to an individual mouse. The model achieves complete discrimination of the 3 groups. (**C**) Correlations of each lipid mediator with the 2 linear discriminant functions are shown on the bottom *x* and left *y* axes. Group centroids for the 2 discriminant functions are plotted on the top *x* and right *y* axes to show the directionality and association of each lipid with the 3 treatment groups. *n* = 7–9 mice per group.

We tested whether a fish oil–enriched diet could modulate the local bioactive lipid mediator metabolome and assessed the functional consequences of regulating tissue levels of these potent mediators in a neutrophillic model of arthritis triggered by transfer of arthritogenic serum (12). This model of arthritis is dependent upon IgG, FcγRs, complement, leukotriene B_4_ (LTB_4_), and IL-1β and is not dependent upon the adaptive immune system, yet requires a variety of innate immune cells — including mast cells, macrophages, and neutrophils — allowing the study of the innate immune system in arthritis (12). Mice fed omega-3–enriched chow exhibited a reduced arthritic score as compared with those on a standard chow diet (Figure 1A). We next performed lipid mediator metabololipidomics on arthritic paws. Mediators from the arachidonic acid (AA), eicosapentaenoic acid (EPA), and docosahexaenoic acid (DHA) bioactive metabolomes were identified and quantified in murine paws in accordance with published criteria, including matching retention times (RTs) on liquid chromatography (LC) and tandem mass spectrometry (MS/MS) fragmentation spectra (ref. 13 and Table 1).

Distinct profiles of lipid mediators were identified in naive, arthritic, and omega-3–supplemented mice undergoing arthritis; lipid mediators could be separated utilizing linear discriminant analysis (Figure 1B). Thus, correlations between individual lipid mediators and the 3 treatment groups were plotted (Figure 1C). Higher levels of MaR1 were found in naive paws as compared with arthritic paws. Comparatively, arthritic joints were associated with higher levels of proinflammatory prostanoids including thromboxane (TX) B_2_, prostaglandin (PG) D_2_, PGE_2_, and PGF_2α_, as well as leukotriene (LT) B_4_. Whereas the paws of arthritic mice supplemented with an omega-3–enriched diet were characterized by elevated levels of SPM, including EPA-derived RvE2 and RvE3 and DHA derived Rvs, of which RvD1 and RvD5 were significantly increased (Table 1 and Figure 1C). These findings suggest that local production of SPM within the joints of omega-3–supplemented mice may contribute to the reduction in arthritis severity.

### Identification of SPM in human RA synovial fluids.

**Figure 2:**
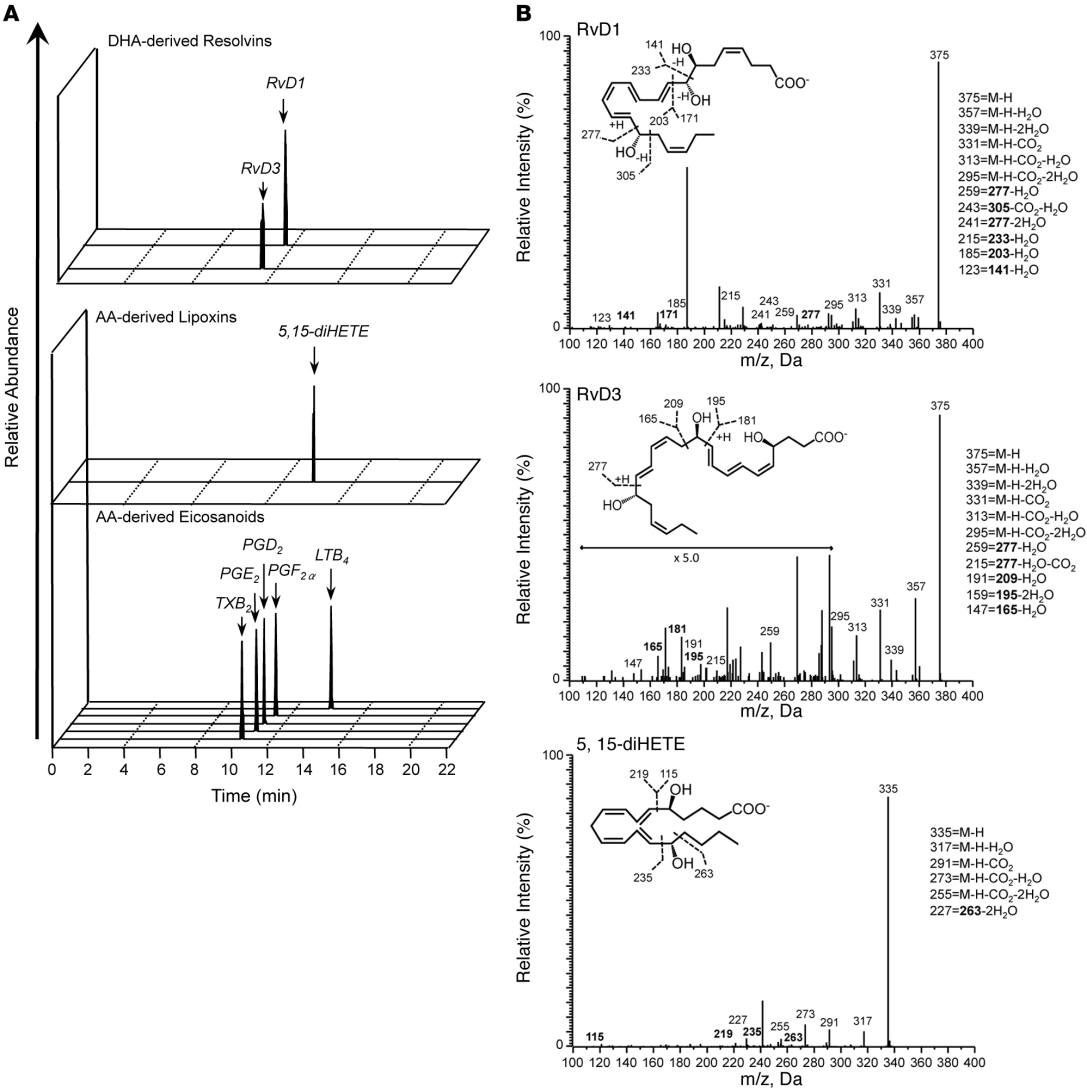
Identification of resolvin D1 (RvD1) and RvD3 in synovial fluids from rheumatoid arthritis (RA) patients. Lipid mediator levels were assessed following solid phase extraction by liquid chromatography tandem mass spectrometry–based (LC-MS/MS–based) metabololipidomics (see Methods for details). (**A**) Representative multiple reaction monitoring (MRM) traces for the identified lipid mediators in human RA synovial fluids (from *n* = 4). (**B**) Accompanying MS/MS spectra utilized for identification. Refer to Table 2 for patient demographics and Table 3 for quantification of bioactive lipid mediators.

We next profiled synovial fluid from human RA patients to determine the bioactive lipid mediator metabolome found within human joints (refer to Table 2 for patient demographics/treatment regimes). Using LC-MS/MS–based lipid mediator metabololipidomics, we identified mediators from all 3 major bioactive metabolomes in human synovial fluids including DHA-derived Rvs and AA-derived eicosanoids (Figure 2A). Similarly to murine arthritic joints, AA-derived autacoids classically associated with joint pain and inflammation were detected within the synovial effusate, including PGE_2_, PGD_2_, PGF_2α_, TXB_2_, and LTB_4_. In these fluids, we also identified the pathway marker for the protective lipoxin family 5,15-diHETE, as well as the D-series Rvs, RvD1 and RvD3. All of these mediators were identified in accordance with published criteria including matching RTs in LC (Figure 2A) and characteristic/diagnostic MS/MS fragmentation patterns (Figure 2B). Of note, the levels of these proresolving mediators in these inflammatory exudates were detected within physiologically relevant concentrations (RvD1, ~31 pM, bioactive concentrations: 10 pM–100 nM; RvD3, ~23 pM, bioactive concentrations: 1 pM–10 nM) (refs. 8, 14, 15, and Table 3).

### Proresolving lipid mediators directly impact neutrophil migration.

As arthritic synovial exudates contain abundant neutrophils, we utilized microfluidic chambers that provide defined spatiotemporal concentration gradients to study the role of SPM in regulating neutrophil chemotaxis in real time and at a single cell level. We directly compared the actions of lipoxin A_4_ (LXA_4_), LXB_4_, RvD1, or RvD2 on neutrophil migration toward IL-8, a classical neutrophil chemotaxis signal. Initially, neutrophils were exposed to IL-8 (10 nM) alone, then exposed to a set concentration (1 nM) of SPM concomitantly with the IL-8 gradient over the subsequent 15 minutes. In the absence of IL-8 superfusion, any captured PMN remained rounded and rapidly detached during the first minute of flow (Supplemental Figure 1A, still images); this was also the case following exposure to SPM alone (data not shown).

**Figure 3:**
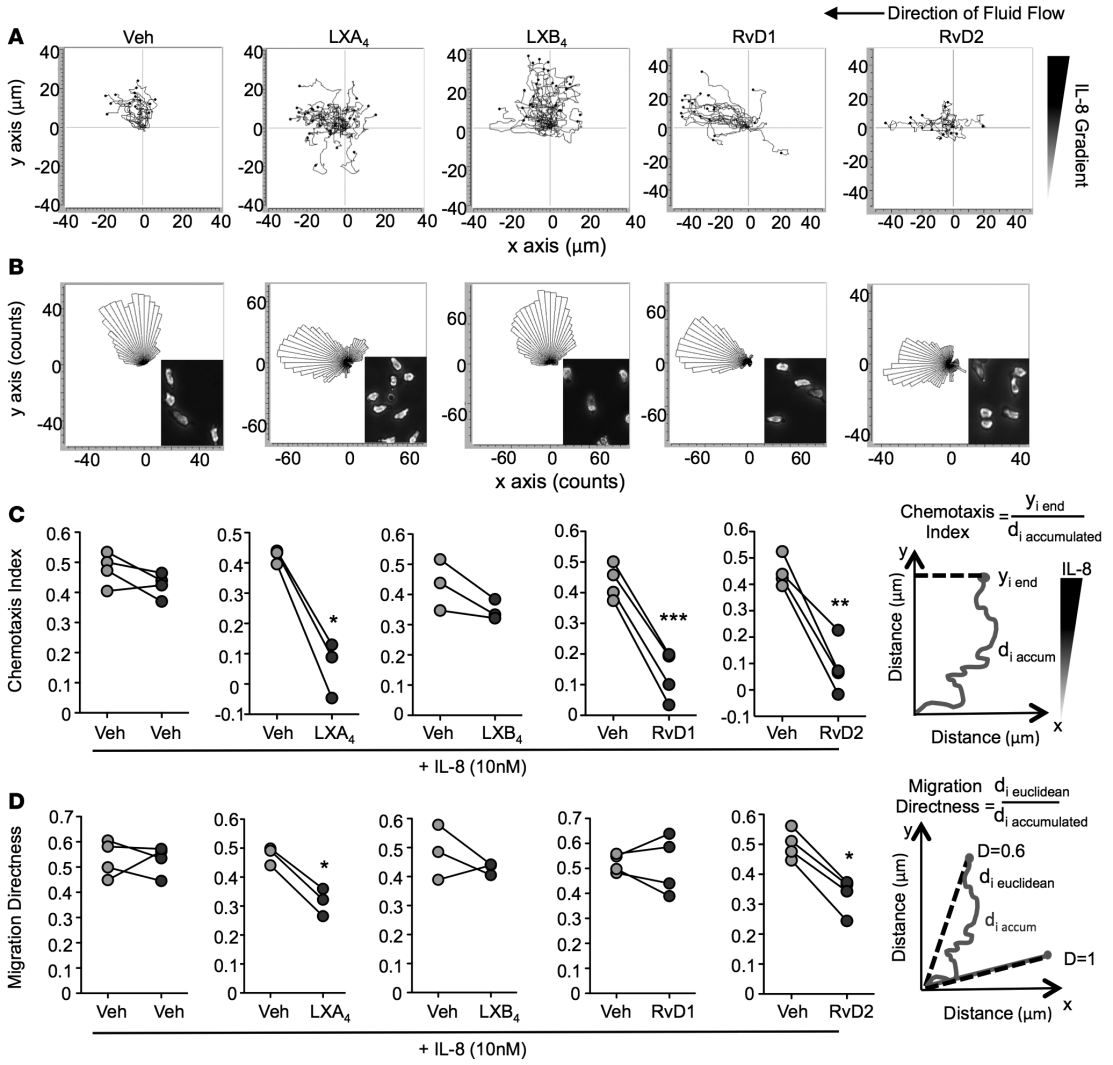
Direct actions of RvD1, RvD2, LXA_4_, and LXB_4_ on human neutrophil chemotaxis. Neutrophils were captured from whole blood of healthy volunteers on P-selectin and ICAM-1–coated microfluidics chambers. Chemotaxis toward an IL-8 gradient (10 nM) was monitored in real time over 30 minutes. During the first 15 minutes, cells were exposed to HBSS + IL-8 (0–10 nM vertical gradient). Then, cells were exposed to a set concentration of resolvin D1 (RvD1), RvD2, lipoxin A_4_ (LXA_4_), LXB_4_ (1 nM), or vehicle (HBSS + 0.1% ethanol) together with the IL-8 gradient over the subsequent 15 minutes. (**A**) Representative trajectory paths and (**B**) rose plots of migrating cells are shown. Insets in **B** show morphology of neutrophils after exposure to IL-8 or proresolving mediators. (**C**) Quantification of neutrophil chemotaxis index and (**D**) directness of neutrophil migration as depicted by the graphs; *n* = 3–4 donors per compound. *P < 0.05, **P < 0.01, and ***P < 0.001 with 2-tailed paired Student’s *t* tests.

Displacement and directionality were determined for each individual cell by tracking the cell centroid utilizing ImageJ software analysis. Following exposure to IL-8, cells migrated in the direction of the chemotactic gradient as seen by vertical cell trajectory paths (Figure 3A; veh), with general direction depicted by the rose plot (Figure 3B; veh). The inset in Figure 3B shows the polarized morphology of neutrophils after exposure to IL-8 plus vehicle (0.1% ethanol). In the presence of LXA_4_, RvD1, or RvD2, neutrophils no longer responded to the chemotactic signal and instead migrated in the direction of fluid flow (Figure 3, A and B). With 1 nM LXB_4_, cells still responded to IL-8 and retained their polarized morphology. Rose plots for each individual donor were plotted separately (Supplemental Figure 1, B–F). The chemotaxis index was calculated for each SPM, as graphically illustrated in Figure 3C. LXA_4_, RvD1, and RvD2 all significantly blunted PMN chemotaxis by approximately 75%. The directionality of neutrophil migration was also calculated for each SPM (Figure 3D); exposure to LXA_4_ or RvD2 significantly reduced direct travel (i.e., increased random movement). These results demonstrate that, at low nanomolar concentrations, LXA_4_, RvD1, and RvD2 each selectively impact and reduce directional migration toward IL-8. We subsequently selected RvD1, which significantly attenuated PMN chemotaxis yet had little effect on random migration, to test in a neutrophilic model of arthritis.

### Treatment of mice with 17R-RvD1 confers protection from inflammatory arthritis.

**Figure 4:**
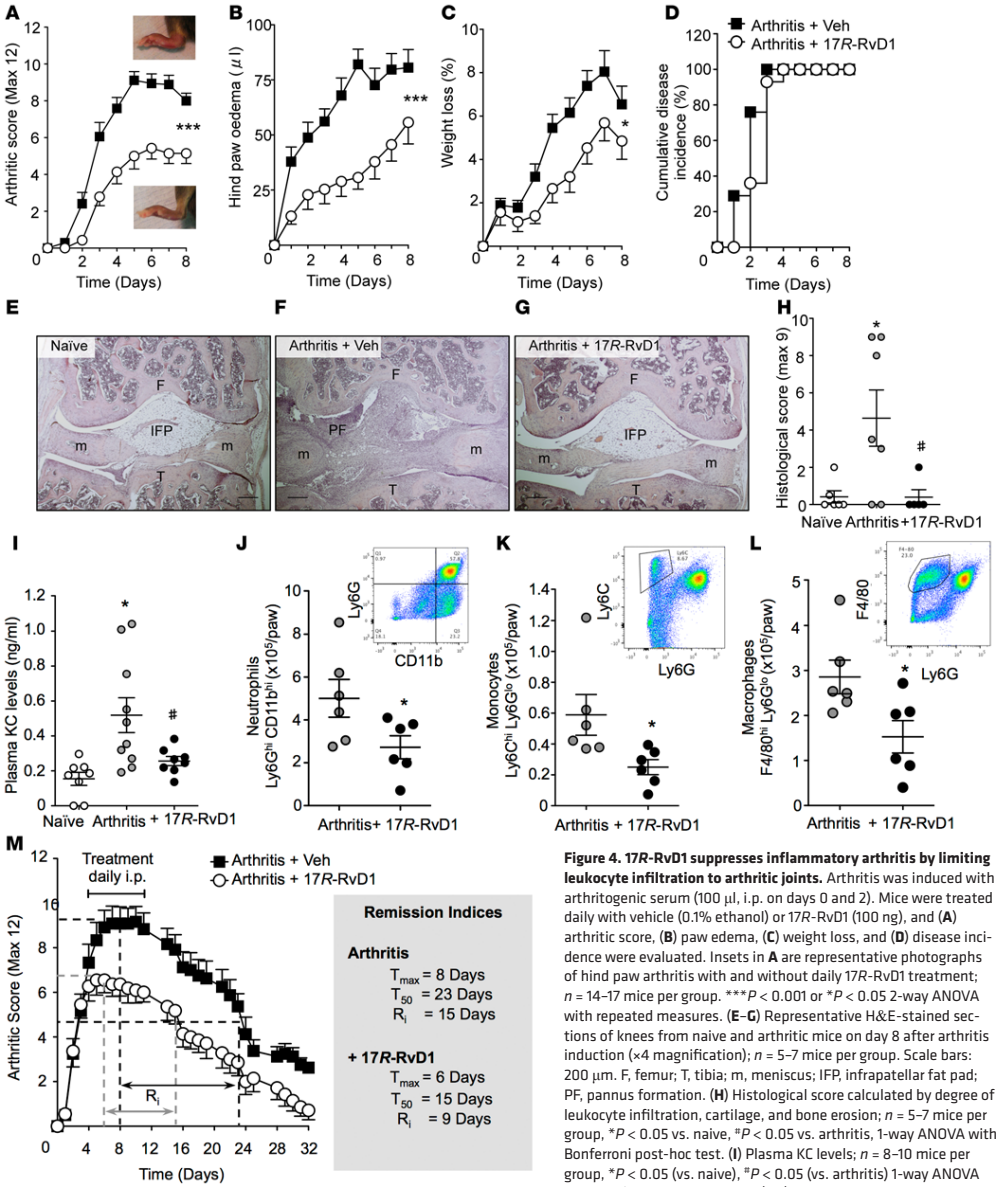
17*R*-RvD1 suppresses inflammatory arthritis by limiting leukocyte infiltration to arthritic joints. Arthritis was induced with arthritogenic serum (100 μl, i.p. on days 0 and 2). Mice were treated daily with vehicle (0.1% ethanol) or 17*R*-RvD1 (100 ng), and (**A**) arthritic score, (**B**) paw edema, (**C**) weight loss, and (**D**) disease incidence were evaluated. Insets in **A** are representative photographs of hind paw arthritis with and without daily 17*R*-RvD1 treatment; *n* = 14–17 mice per group. ***P < 0.001 or *P < 0.05 2-way ANOVA with repeated measures. (**E**–**G**) Representative H&E-stained sections of knees from naive and arthritic mice on day 8 after arthritis induction (×4 magnification); *n* = 5–7 mice per group. Scale bars: 200 μm. F, femur; T, tibia; m, meniscus; IFP, infrapatellar fat pad; PF, pannus formation. (**H**) Histological score calculated by degree of leukocyte infiltration, cartilage, and bone erosion; *n* = 5–7 mice per group, *P < 0.05 vs. naive, ^#^*P* < 0.05 vs. arthritis, 1-way ANOVA with Bonferroni post-hoc test. (**I**) Plasma KC levels; *n* = 8–10 mice per group, *P < 0.05 (vs. naive), ^#^*P* < 0.05 (vs. arthritis) 1-way ANOVA with Bonferroni post-hoc test. (**J**–**L**) Arthritic paws were digested to liberate leukocytes; cells were counted by light microscopy and leukocyte subsets defined using specific antibodies by flow cytometry; *n* = 6 mice per group, *P < 0.05 unpaired Student’s *t* test. (**M**) Mice were treated daily following overt signs of arthritis (day 4) for 1 week with vehicle (0.1% ethanol) or 17*R*-RvD1 (100 ng), and arthritic score was evaluated over 32 days. Arthritis remission indices were calculated; *n* = 11–12 mice per group.

RA is a progressive inflammatory disease characterized by extensive infiltration of leukocytes into the synovial cavity and articular tissues. Given that RvD1 significantly regulated human neutrophil responses and its 17*R*-epimer retains the bioactions of RvD1 while displaying higher metabolic stability (11), we next investigated the therapeutic potential of 17*R*-RvD1 (7*S*,8*R*,17*R*-trihydroxy-4*Z*,9*E*,11*E*,13Z,15*E*,19*Z*-docosahexaenoic acid) in protecting against leukocyte-mediated join damage. Mice were injected with arthritogenic K/BxN serum and monitored over 8 days. Administration of K/BxN serum triggered robust arthritis, which rapidly developed over the first few days (Figure 4A). 17*R*-RvD1–treated mice exhibited a lower clinical score and reduced hind paw edema compared with vehicle-treated mice (Figure 4, A and B). 17*R*-RvD1 also protected mice from the transient weight loss associated with arthritis (Figure 4C). Disease penetrance, defined as the proportion of mice developing a clinical score greater than 1 out of a maximum 12, was 100% regardless of treatment group (Figure 4D), yet the clinical data indicates 17*R*-RvD1 significantly reduced disease severity.

In addition to the macroscopic assessment of arthritis, histological analyses were performed on knee joints to assess inflammatory infiltrate, cartilage degradation, and evidence of bone erosion. Microscopic analyses of the naive, nonarthritic articular joint presented little evidence for leukocyte infiltration and demonstrated intact cartilage and bone. Arthritic serum provoked a marked accumulation of leukocytes within the joint, as well as evident pannus formation and synovial hypertrophy as compared with naive animals, whereas 17*R*-RvD1 treatment limited leukocyte recruitment, synovitis, and pannus intrusion (Figure 4, E–G, and Supplemental Figure 2, A and B). Blinded analyses of histopathological sections revealed a significant tissue protection by 17*R*-RvD1 (Figure 4H). Levels of KC (CXCL1), a key chemokine known to drive inflammatory arthritis and promote leukocyte recruitment, were assessed (16). Plasma levels were elevated during arthritis and were significantly dampened following 17*R*-RvD1 treatment (Figure 4I). Further studies were performed to decipher which leukocytes infiltrated arthritic joints. Flow cytometric analyses identified neutrophil (Ly6G^hi^CD11b^hi^), monocyte (Ly6C^hi^Ly6G^lo^), and macrophage (F4/80^hi^Ly6G^lo^) accumulation within arthritic paws. Each of these leukocyte subtypes was significantly reduced in paws from 17*R*-RvD1–treated mice (Figure 4, J–L).

Current arthritis treatment strategies aim to intervene early to reduce synovitis and prevent cartilage degradation and bone erosion (3). Hence, our interest was to determine whether 17*R*-RvD1 would be effective at reducing the clinical signs of early experimental arthritis. Therapeutic administration of Rvs at the peak of an acute inflammatory response is known to accelerate the resolution process (17). Therefore, the time taken to remit (remission interval) based on a clinical score was calculated analogously to the resolution interval widely used to calculate the loss of neutrophils from an inflammatory site by 50% (17, 18). 17*R*-RvD1 was tested as a therapeutic agent after the second dosing of K/BxN serum when mice exhibited overt signs of arthritis (100 ng, i.p. daily from day 4–10). Rv treatment limited the arthritis severity (T_max_; veh day 8, max. score 9.5 vs. 17*R*-RvD1 day 6, max. score 7) and shortened the remission interval (R_i_; veh 15 days, 17*R*-RvD1 9 days) (Figure 4M). This experiment demonstrates the potential of 17*R*-RvD1 as a therapeutic for limiting tissue damage in patients with early RA.

### Administration of 17R-RvD1 selectively modulates the local bioactive lipid metabolome within arthritic paws.

**Figure 5:**
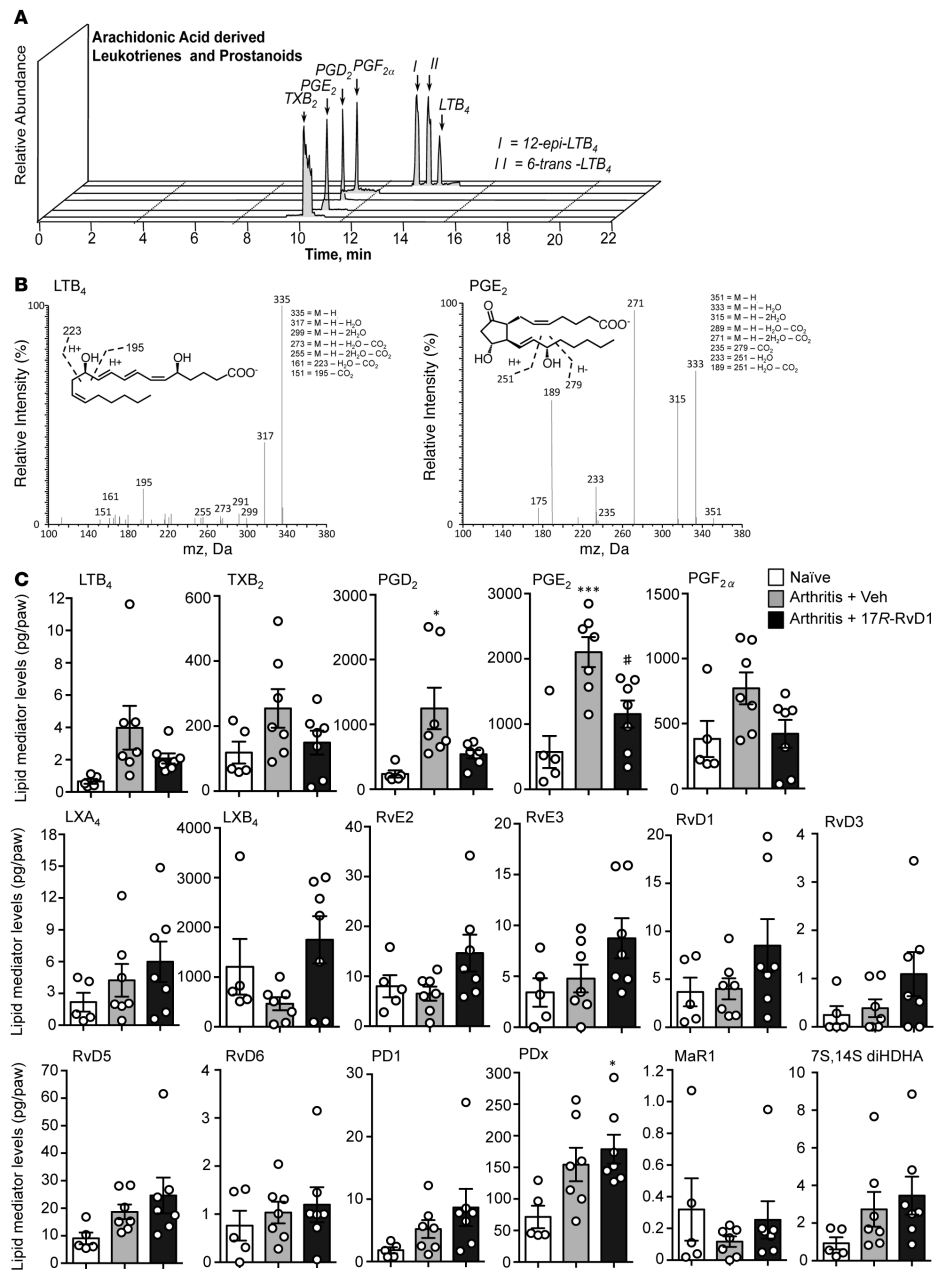
17*R*-RvD1 treatments modulate the local production of bioactive lipid mediators within inflamed paws. Arthritic mice were treated daily with vehicle (0.1% ethanol) or 17*R*-RvD1 (100 ng), and paws were collected for metabololipidomics analysis on day 8; naive mice were used as controls. (**A**) Relative abundance of key arachidonic acid–derived leukotrienes and prostanoids. Representative mass spectrum of (**B**) LTB_4_ and PGE_2_ within arthritic paws. (**C**) Absolute quantification of lipid mediator levels in arthritic paws identified using scheduled multiple reaction monitoring (MRM). *n* = 5–7 per group. *P < 0.05 and ***P < 0.001 vs. naive and ^#^*P* < 0.05 vs. arthritis with 1-way ANOVA and Bonferroni post-hoc test.

Because RvD1 regulates proinflammatory eicosanoid biosynthesis during acute peritonitis (15), we next performed lipid mediator metabololipidomics on arthritic paws obtained 8 days after K/BxN serum administration to elucidate the potential mechanism(s) underlying 17*R*-RvD1 actions. Identification was conducted using established criteria (13), including LC RT and at least 6 fragment diagnostic ions in the MS/MS as illustrated for LTB_4_ and PGE_2_ (Figure 5, A and B). In these paws, mediators from each of the 3 major bioactive metabolomes were identified, including the following eicosanoids: LTB_4_, LXA_4_, and LXB_4_ from the lipoxygenase pathway and PGE_2_, PGD_2_, PGF_2α_, and TXB_2_ from the cyclooxygenase pathways (Figure 5C). Multiple reaction monitoring (MRM) was utilized to quantify the individual mediator amounts. We found that 17*R*-RvD1 downregulated production of proinflammatory LTB_4_, an essential mediator required for the development of experimental arthritis (19), along with TXB_2_ and PGE_2_, PGD_2_, and PGF_2α_ while activating protective LXA_4_ and LXB_4_ biosynthesis (Figure 5C). We also found that 17*R*-RvD1 stimulated the biosynthesis of EPA-derived SPM including the E-series Rvs RvE2 and RvE3 by approximately 50%. From the DHA bioactive metabolome, protectin Dx (PDx) was significantly elevated in mice treated with 17*R*-RvD1. Thus, this regulation of lipid mediators may contribute to the joint protective actions following pharmacological delivery of 17*R*-RvD1.

**Figure 6:**
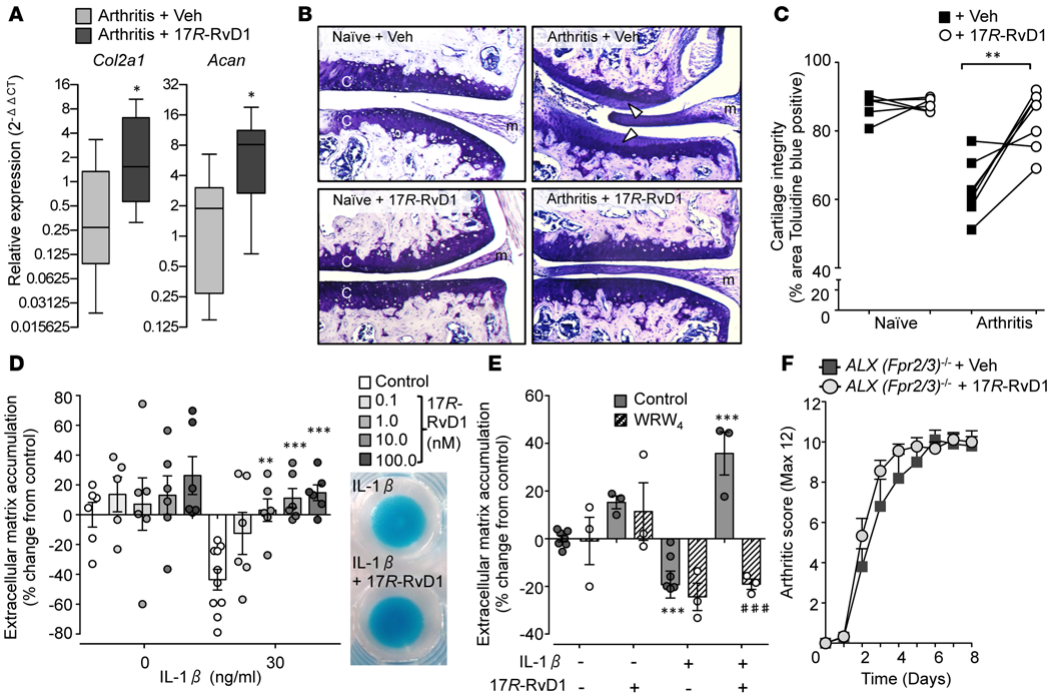
Intraarticular treatment with 17*R*-RvD1 protects from cartilage degradation during inflammatory arthritis. (**A**) Arthritis was induced with arthritogenic serum (100 μl, days 0 and 2), mice were treated daily with vehicle (0.1% EtOH) or 17*R*-RvD1 (100 ng, i.p.), and paws were collected for gene expression analysis on day 8 (see Methods); *n* = 8–9 mice per group, *P < 0.05 with Mann-Whitney *t* test. Col2a1, collagen type II α 1; Acan, aggrecan. (**B** and **C**) Mice received arthritogenic serum (100 μl, i.p. days 0 and 2) and were treated locally on day 3 with vehicle (left knee; 5 μl PBS containing 0.1% EtOH) or 17*R*-RvD1 (right knee; 5 μl, 100 ng 17*R*-RvD1). On day 5, knee joints were collected and stained with toluidine blue; *n* = 7 mice per group. Naive mice were administered vehicle or 17*R*-RvD1 locally, and knees collected after 2 days; *n* = 6 mice per group. (**B**) Representative images (×20 magnification) of histological sections from naive and arthritic joints are shown. C, cartilage; m, meniscus. Loss of glycosaminoglycans indicated by arrow heads. (**C**) Cartilage integrity calculated from percentage area of cartilage positive for toluidine blue staining; **P < 0.01 with 2-tailed paired Student’s *t* test. (**D** and **E**) In vitro analyses of chondroprotection utilizing human C28/I2 micromasses. (**D**) Micromasses were treated with or without IL-1β (30 ng/ml) alone or with 17*R*-RvD1 (0.1–100 nM) and ECM accumulation evaluated (see Methods); *n* = 6–10 per group, **P < 0.01, ***P < 0.001 vs. IL-1β with 2-way ANOVA and Bonferroni post-test. Inset representative micromasses stained with Alcian blue prior to dye extraction. (**E**) FPR2/ALX receptor antagonist WRW_4_ (10 μM) was added to micromasses 10 minutes prior to 17*R*-RvD1 and ECM accumulation evaluated after 24 hours; *n* = 3–7 per group, ***P < 0.001 vs. vehicle, ^###^*P* < 0.001 vs. respective control with 2-way ANOVA and Bonferroni post-test. (**F**) Dependency of *Fpr2/3* (ALX) receptor for 17*R*-RvD1 protection from inflammatory arthritis. Arthritis was induced in *Fpr2/3-*null (ALX-null) mice (see Methods), mice were treated daily with vehicle (PBS with 0.1% EtOH) or 17*R*-RvD1 (100 ng, i.p.), and arthritic score was assessed; *n* = 9–10 mice per group.

Gene expression analysis was performed on paw tissue from arthritic mice treated with vehicle and 17*R*-RvD1 observing selective modulatory functions. A trend for downmodulation of *Il1b*, the key cytokine driving K/BxN arthritis; *Ly6g*, a marker of neutrophil influx; and *Ptsg2* (cyclooxygenase-2), essential for PG biosynthesis, was detected in mice given 17*R*-RvD1. On the other hand, higher expression of *Alox15* (15-lipoxygenase) transcript, a key gene in SPM biosynthesis, was detected in mice treated with 17*R*-RvD1 (Supplemental Table 1). Most notably, a significant upregulation of key genes involved in cartilage matrix synthesis was observed, namely type II collagen and aggrecan (Figure 6A). This unpredicted finding brought us to investigate the impact of 17*R*-RvD1 treatments on cartilage erosion during K/BxN arthritis. After the onset of arthritis (day 3), mice were administered 17*R*-RvD1 (100 ng) directly into the knee joint, along with PBS (+0.1% ethanol) into contralateral knees as control, with histological analyses being performed on day 5. Cartilage integrity was first assessed in naive mice, whereby the cartilage remained intact regardless of PBS or 17*R*-RvD1 treatment, as measured by toluidine blue staining (Figure 6B, left panels, and Figure 6C analyses). However, a marked reduction in toluidine blue staining on the cartilage surface was observed in PBS-injected knees of arthritic mice, indicating loss of glycosaminoglycans (Figure 6B, upper right panel, white arrowheads). Remarkably, a significant protection from cartilage damage was evident in 17*R*-RvD1–treated joints as compared with paired contralateral control knees (Figure 6B, lower right panel, and Figure 6C, analyses).

To determine whether this was a direct effect on the chondrocytes rather than an indirect reduction of leukocyte/stromal cell–induced cartilage degradation, we took advantage of the human chondrocyte (C28/I2) micromass system to evaluate extracellular matrix (ECM) accumulation (20). Treatment of micromasses with 17*R*-RvD1 (0.1–100 nM) resulted in a slight but nonsignificant increase in ECM accumulation (Figure 6D). When chondrocytes were stimulated with IL-1β, a characteristic loss in ECM was quantified. Of note, 17*R*-RvD1 treatments significantly protected against the catabolic effects of concomitant IL-1β stimulation, leading to increased ECM deposition (Figure 6D and representative images of Alcian blue stained micromasses). As chondrocytes express the 17*R*-RvD1 receptor ALX/FPR2 (21) and 17*R*-RvD1 demonstrated direct actions on chondrocytes, we sought to determine whether this was via receptor ligation of ALX/FPR2. Addition of the specific antagonist WRW_4_ did not have any effect on resting or IL-1β–treated micromasses alone. However, the accumulative effects of 17*R*-RvD1 were reversed by the addition of WRW_4_, suggesting this protective effect was mediated via ALX/FPR2 (Figure 6E). Further support of ALX/FPR2 receptor dependency for the protective actions of 17*R*-RvD1 was attained in vivo by utilizing mice nullified with the orthologue receptor *Fpr2/3* (22). Accordingly, we subjected these mice to arthritis and treated them daily with either vehicle (PBS + 0.1% ethanol [EtOH], i.p.) or 17*R*-RvD1 (100 ng, i.p.). In the absence of *Fpr2/3*, 17*R*-RvD1 no longer attenuated the clinical score (Figure 6F), the swelling of hind paws, or the arthritis-related manifestation of cachexia, and disease penetrance was almost identical between the 2 genotypes (data not shown). Histopathology of murine hind paws indicated substantial leukocyte infiltration (Supplemental Figure 3A), with near maximal score (3/3) for both genotypes (Supplemental Figure 3B). In summary, joint protection afforded by 17*R*-RvD1 was abolished in the absence of ALX/*Fpr2/3*.

## Discussion

RA is a chronic progressive autoimmune disease characterized by inflammation of the joints that manifests as swelling, pain, and functional impairment. This condition can also lead to muscle wasting and osteoporosis and is associated with systemic inflammatory comorbidities such as periodontitis and cardiovascular disease (1, 23). Although current therapeutics have revolutionized the management of RA, existing treatments aimed at limiting joint inflammation are associated with unwanted side effects including gastrointestinal (GI) disturbances and increased risk of infections. In addition, although current therapeutics may lead to significant improvement in patient prognosis by slowing or stopping disease progression, treatment rarely repairs joint damage (5).

We report here an elevated local production of SPM in arthritic joints from mice supplemented with omega-3–enriched chow, a biochemical response that correlates with a reduction in arthritis severity. Using targeted lipid mediator metabololipidomics, we identified RvD1 as an SPM that was both elevated in arthritic paws from omega-3–supplemented mice and present in human RA synovial fluids, detected within the concentration range for physiological activities. Thus, when we tested the stable epimer 17*R*-RvD1 (11), a selective modulation of local lipid mediator biosynthesis and reduction in joint leukocyte infiltration was observed paralleled by macroscopic amelioration of arthritis severity, cachexia, and hind-paw edema. We also uncovered tissue-protective functions of 17*R*-RvD1, such as stimulation of chondrocyte matrix production and protection from cartilage degradation. Together, these findings identify 17*R*-RvD1 as a prototype for new therapeutic approaches for the treatment of arthritis.

Timely resolution of an inflammatory response is pertinent to maintain tissue homeostasis. To date, we and others have identified a number of endogenous mediators that activate resolution pathways to actively switch off inflammation, including annexin-A1 (24), gaseous mediators (e.g., hydrogen sulphide, ref. 25; and carbon monoxide, refs. 26, 27) as well as SPM (lipoxins, Rvs, protectins, and MaRs; refs. 2, 7). Dysregulated inflammation is a common determinant of many pathologies, yet it was only recently appreciated that diseases such as RA may persist, at least in part, due to a failure of resolution (28). Accordingly, essential protective roles for endogenous SPM and their receptors are indicated in experimental arthritis. Null mice lacking either 12/15-LOX (a key enzyme involved in SPM biosynthesis) or ALX/FPR2 (a high-affinity receptor for RvD1, LXA_4_, and annexin-A1), display exacerbated disease severity and tissue damage in inflammatory arthritis (29, 30). Additionally, mice nullified for 5-LOX (another key enzyme involved in SPM biosynthesis) exhibit intensified and prolonged duration of infectious arthritis (31). Furthermore, inhibiting COX-2 activity delays the resolution of collagen-induced arthritis, which can be restored with PGE_2_-mediated LXA_4_ production (32). Together, results from these studies emphasize the importance of endogenous SPM for the control of arthritis. Early studies demonstrated the protective effects of supplementing mice with fish oil–enriched diet, with reduced susceptibility and lower severity scores when subjected to collagen-induced arthritis (33, 34). Congruently, we found that arthritic mice fed a fish oil–enriched diet displayed attenuated arthritis that was associated with a reduction in proinflammatory lipid mediator levels and an enhanced biosynthesis of omega-3–derived SPM.

Enzymes of the SPM biosynthetic pathway, as well as the RvD1 receptor, are expressed in the rheumatoid synovium. Compared with osteoarthritis (OA) synovia, elevated levels of 5- and 15-LOX, as well as ALX/FPR2, are detected in synovial tissue of RA patients (35, 36). In the present study, we identified both RvD1 and RvD3, along with the lipoxin pathway marker 5,15-diHETE, in synovial fluids from RA patients. These results add to the recent identification of RvD5, MaR1, and LXA_4_ within synovial fluid (37). From these findings, we propose that metabololipidomic profiling can assist in RA patient stratification with the definition of lipid mediator profiles associated with specific cohorts and in the assignment of precision medical treatments and personalized RA patient care.

Aberrant infiltration of immune cells into the joint and the subsequent destruction of bone and cartilage drive loss of function. During active phases of RA, neutrophils present within synovial fluids contribute to disease pathogenesis via release of cytotoxic enzymes and reactive oxygen species (38). We previously reported that RvD1 (0.1–100 nM) significantly dampened neutrophil recruitment to TNF-α stimulated endothelium (39), a pivotal cytokine that drives RA (6). Using microfluidics chambers to study bioactions at the single cell level under flow of continuous chemokine gradients, we observed potent actions of RvD1 at low nanomolar concentrations, blunting directed neutrophil locomotion. Thus, we utilized the K/BxN serum transfer model of arthritis to test the efficacy of the stable epimer 17*R*-RvD1. In this model of symmetric polyarthritis, daily treatment with 17*R*-RvD1 preserved joint integrity and lessened the histopathology of arthritis, with reduced synovitis and pannus intrusion and overall leukocyte infiltration. We previously demonstrated that 17*R*-RvD1–enriched nanoparticles limit leukocyte recruitment into the inflamed temporomandibular joint (40). Our current results emphasize the protective actions of 17*R*-RvD1 in limiting joint inflammation.

It is known that a lipid-cytokine-chemokine cascade drives neutrophil recruitment during experimental arthritis (16). Mechanistically, we found that administration of 17*R*-RvD1 downregulated *Ptsg2* and elevated *Alox15* gene expression, resulting in the selective modulation of the local lipid mediator metabolome. We measured reduced LTB_4_ and PG levels that contribute to the pain and perpetuation of joint inflammation and identified select regulation of SPM levels that may contribute to the protective effects afforded by 17*R*-RvD1 administration. We also determined that 17*R*-RvD1 reduced systemic KC (CXCL1) levels, a principal chemokine that recruits neutrophils, and downregulated *Il1b* transcripts, a key cytokine that amplifies arthritis. The main cellular source of this cytokine in this experimental model of arthritis is neutrophils (16), and correspondingly, we observed a reduction in neutrophil *Ly6g* transcripts. The antiarthritic actions of 17*R*-RvD1 were lost in *Fpr2/3*-null mice, reiterating this essential signaling pathway for RvD1 in murine inflammation (15, 41).

Once damaged, articular cartilage has limited intrinsic capability to self-repair. Importantly, we uncovered unique tissue protective functions of 17*R*-RvD1 in maintaining cartilage integrity during inflammatory arthritis. Chondrocytes were identified as target cells for the protective actions of RvD1, stimulating ECM deposition. We provide evidence that 17*R*-RvD1 protects chondrocytes from IL-1β–induced degradation via direct ALX/FPR2 receptor ligation. This finding complements the array of known functions that proresolving mediators exert to repair injured tissue in the context of wound healing (42), tissue regeneration (43, 44), and restitution of gut epithelial barrier function (45). Our results indicate that mediators of resolution could be modeled to repair cartilage if, and when, their receptor target is presented on the chondrocyte. Recent work indicates that ALX/FPR2 is expressed by human chondrocytes and surface expression is upregulated in catabolic settings, like those evoked with IL-1β (21).

The results we present here using synthetic RvD1 as a prototype therapeutic tool from the SPM portfolio, a choice dictated by detection of this bioactive mediator in mouse and human joints, opens the opportunity to exploit this line of research for innovative therapeutic strategies in RA that combine potent antiinflammatory with tissue-protective properties. Modeling RvD1 analogues or agonists at their receptor provide another avenue for therapeutic development (24). It is important to point out the prediction that, compared with current therapeutic options, RvD1 and — in general — SPM-based therapies will be beneficial in multiple respects. Firstly, SPM do not provoke immune suppression (a major side effect associated with biologics, e.g., anti-TNF therapy; ref. 6) and rather stimulate host phagocyte functions to enhance phagocytosis and killing of microorganisms (8, 46, 47). Secondly, SPM exert bone-sparing properties (a major secondary effect associated with long-term glucocorticoid therapy is bone loss). In the context of experimental periodontitis, RvE1 prevents alveolar bone loss (48), accelerates bone regeneration (43), and inhibits osteoclast maturation and bone resorption (49). Additionally, parathyroid hormone stimulates RvD1 and RvD2 to enhance macrophage efferocytosis of apoptotic osteoblasts, highlighting a role for SPM in bone homeostasis (50). Thirdly, SPM are potent analgesics (51), and relevantly, 17*R*-RvD1 is antihyperalgesic in a model of arthritic pain (52). Finally, with the present study, we demonstrate chondroprotective functions of SPM, combating catabolic stimulation and promoting direct anabolic responses enabling protection from cartilage degradation during inflammatory arthritis.

In summary, we provide new mechanisms underlying the antiarthritic properties evoked by omega-3 consumption in arthritis (10) and focus on a specific omega-3–derived SPM, namely RvD1 and its stable epimer 17*R*-RvD1, to establish proof-of-concept for the validity of this approach. We defined bioactions for this proresolving endogenous lipid mediator in the context of joint inflammation and chondroprotection. Together, these results give the foundation for the development of innovative therapeutic strategies to avert joint destruction during RA with limited side effects, ultimately for the benefit of the patients.

## Methods

### Animals.

Male 12-week-old, approximately 30 g, C57Bl/6 (Charles River Laboratories) or *Fpr2/3*-null (ALX-null) mice (generated on a C57Bl/6 background as described, and back-backcrossed 10 times; ref. 22) were maintained on a standard chow pellet diet and had access to water ad libitum, with a 12-hour light-dark cycle. For omega-3 diet-enrichment studies, mice were maintained on a CRM (P) plus 10% salmon oil or standard CRM (P) chow (Special diet services, LBS Biotech) 3 weeks prior to and throughout the duration of experiments.

### Real-time neutrophil chemotaxis using microfluidics chambers.

Microfluidics chambers were engineered for testing the actions of bioactive lipid mediators on the chemotactic behavior of human neutrophils as described previously (53). The main channel for assessing neutrophil chemotactic behavior was modified by physical adsorption of P-selectin (50 μg/ml, R&D Systems) and ICAM-1 (10 μg/ml, R&D Systems) for 30 minutes, followed by blocking with 2% human serum albumin (HSA, Sigma-Aldrich) in HBSS (Sigma-Aldrich) immediately before use. Steady-state gradients of IL-8 (0–10 nM, R&D Systems) were formed in the 2 gradient generators connected to the main channel, one containing vehicle (0.1% ethanol) and the other containing an overlaying uniform concentration (1 nM) of the specialized proresolving lipid mediator for testing (LXA_4_, LXB_4_, RvD1, or RvD2; Cayman Chemicals). Approximately 10 μl of capillary blood was then collected from healthy volunteers using a BD genie lancet (BD Biosciences), and diluted 1:10 with HBSS, 0.2% HSA. Neutrophils were captured from flowing blood over approximately 3 minutes, and the first valve opened for infusion of the IL-8 gradient (fluid flow removed the majority of RBC and other cells that were not tethered to the coated chamber), allowing direct monitoring of neutrophils captured on the chamber surface. After 15 minutes, the gradient was switched to the second gradient generator containing a uniform concentration of the SPM combined with 10 nM IL-8 gradient. Neutrophil migration in the chemokine gradient and their response(s) to addition of different SPM were recorded every 6 seconds over a 30-minute period with a digital camera and Image Pro Plus software. Cell migration was analyzed using the cell-tracking function in ImageJ software, and tracks were analyzed utilizing Ibidi Chemotaxis and Migration Tool. Only cells that started and remained within the field of view over the entire course of video capture were analyzed. At least 20 cells were tracked per donor, with 3–4 donors per test compound.

### K/BxN serum transfer model of inflammatory arthritis.

K/BxN serum was generated in house by breeding NOD/Shiltj mice (Charles River Laboratories) with KRN transgenic mice (gift from Mohini Gray, Edinburgh University, Edinburgh, United Kingdom); the subsequent offspring (K/BxN mice) express both the T cell receptor (TCR) transgene KRN and the MHC class II molecule Ag7 and spontaneously develop a severe inflammatory arthritis. Serum was collected from K/BxN mice at 10 weeks of age, which causes a similar arthritis when transferred to recipient mice due to autoantibodies recognizing glucose-6-phosphate isomerase (GPI) depositing on the cartilage surface. Serum transfer–induced arthritis was performed by i.p. injection of 100 μl of arthritogenic serum on days 0 and 2 in WT or *ALX/Fpr2/3*-null mice. Disease was monitored by assessing the clinical score (maximum 12 points per animal, 3 per limb with the following scoring system: 0, no evidence of inflammation; 1, inflammation in one of the following aspects: individual phalanges joints, localized wrist/ankle, or swelling on surface of paw; 2, inflammation on two aspects of paw; 3, major swelling on all aspects of paw). Paw edema was assessed by water displacement plethysmometry (Ugo Basile). Mice were weighed daily for signs of cachexia. For prophylactic treatment regime, WT mice were administered vehicle (0.1% EtOH) or 17*R*-RvD1 (100 ng) in 100 μl of saline i.p. daily (from day 0, 5 minutes prior to arthritogenic serum administration). For therapeutic treatment protocol, WT mice were administered vehicle (0.1% EtOH) or 17*R*-RvD1 (100 ng) in 100 μl of saline i.p. daily following overt signs of arthritis (day 4) over a 7-day period. The arthritis remission interval was calculated based on arthritic score and calculated by analogy to the resolution indices widely used to calculate the loss of neutrophils from an inflammatory site (17, 18). At the end of experiments, blood was collected via cardiac puncture into heparinized syringes and centrifuged to obtain platelet-free plasma. For intraarticular treatments, WT mice were administered K/BxN serum on days 0 and 2 (100 μl, i.p.) and were treated locally on day 3 with vehicle (left knee; 5 μl PBS containing 0.1% EtOH) or 17*R*-RvD1 (right knee; 5 μl, 100 ng 17*R*-RvD1), and joints were harvested for histological analysis on day 5.

### Molecular analyses.

Arthritic ankle joints were collected on day 8 and stored in RNAlater at –80°C prior to homogenising using Precellys ceramic beads (Bertin Technologies). RNA was extracted using RNeasy Plus mini kit (Qiagen), and genomic DNA contamination eliminated with Turbo DNA-free kit (Applied Biosystems). Complementary DNA was synthesized using SuperScriptIII reverse transcriptase and OligoDt primers (Invitrogen). Quantitative PCR (qPCR) was performed using QuantiTect primers (Qiagen) and ABI Prism 7900 sequence detector system (Applied Biosystems). Relative expression values were calculated following normalization to endogenous housekeeping gene *Rpl32* and using the 2^−(ΔΔCt)^ method normalized to a naive mouse (calibrator sample).

### Flow cytometry.

Leukocytes were isolated from arthritic paws following tissue digestion. Briefly, paws were collected after 8 days of arthritis, skin was carefully removed, and digestion buffer was added (collagenase D [Roche; 0.5 μg/ml] and DNAse [Sigma-Aldrich; 40 μg/ml] in serum-free RPMI). Paws were incubated at 37°C with gentle agitation, and liberated cells were collected via a 70-μm cell-strainer and kept on ice. Another 15 ml of digestion buffer was added to the paws and incubated for a further 30 minutes. Liberated cells were centrifuged at 400 *g* for 10 minutes and resuspended in PBS for counting. Leukocytes were first stained with Zombie NIR (BioLegend; 1:500, 20 min, 4°C) to identify live cells, and distinct leukocyte subtypes were identified using the following antibodies from eBioscience: CD45 (25 μg/ml, clone 30-F11), CD11b (0.2 μg/ml, clone M1/70), Ly6G (25 μg/ml, clone RB6-8CS), Ly6C (0.4 μg/ml, clone HK1.4), and F4/80 (10 μg/ml, clone BM8). Samples were analyzed using an LSR Fortessa flow cytometer and FlowJo software (Tree Star Inc.).

### Histology.

Joints were decalcified and paraffin embedded. Sections (8 μm) were stained with H&E or 1% aqueous toluidine blue, and standard light microscopy was used to determine the degree of leukocyte infiltration/synovitis/pannus formation, bone erosion, and cartilage damage, which were each graded from 0 (no disease) to 3 (severe); a maximum score of 9 was possible, as averaged by 2 independent examiners, who were blinded to the experiment. Cartilage integrity was calculated from percentage area of cartilage stained with toluidine blue using thresholds applied with ImageJ software.

### Determination of plasma KC (CXCL1) levels.

Mice were sacrificed on day 8 following induction of arthritis, and blood was collected via cardiac puncture into heparinized syringes and centrifuged to obtain platelet-free plasma. KC (CXCL1) values were determined by ELISA.

### Targeted LC-MS/MS–based lipidomics of arthritic paws and synovial fluids.

Paws were collected and immediately transferred to liquid nitrogen prior to homogenization in 1 ml ice cold MeOH containing deuterated internal standards (d_4_-LTB_4_, d_8_-5S-HETE, d_4_-PGE_2_, d_5_-LXA_4_, and d_5_-LTC_4_, 500 pg each) and homogenized using a glass dounce. Synovial fluid (0.5 ml) from RA patients (Dx Biosamples) were placed in 1 ml ice cold MeOH containing deuterated internal standards (d_4_-LTB_4_, d_8_-5S-HETE, d_4_-PGE_2_, d_5_-LXA_4_, and d_5_-RvD2, 500 pg each). All samples were kept at –20°C for 45 minutes to allow for protein precipitation and subjected to solid phase extraction as in ref. 13. Methyl formate fractions were then brought to dryness using a TurboVap LP (Biotage), and products were suspended in water-methanol (50:50 vol:vol) for LC-MS-MS. A Shimadzu LC-20AD HPLC and a Shimadzu SIL-20AC autoinjector (Shimadzu), paired with a QTrap 6500 (AB Sciex) was utilized and operated as described (13). To monitor each LM and respective pathways, an MRM method was developed with diagnostic ion fragments and identification using recently published criteria (13), including matching RT to synthetic and authentic materials and at least 6 diagnostic ions for each LM. Calibration curves were obtained for each using authentic compound mixtures and deuterium labeled LM at 3.12, 6.25, 12.5, 25, 50, 100, and 200 pg. Linear calibration curves were obtained for each LM, which gave r^2^ values of 0.98–0.99.

### Chondrocyte micromass assay.

The human chondrocyte cell line C28/I2 (gift from Mary Goldring, Cornell Medical College, New York, New York, USA; ref. 54) was utilized for micromass assays as described previously (20). Micromasses were stimulated with or without IL-1β (30 ng/ml) alone or in combination with 17*R*-RvD1 (0.1–100 nM) for 24 hours, and ECM accumulation was calculated following staining with Alcian blue as reported (21). Briefly, micromasses were fixed (4% glutaraldehyde, 15 min) acidified with HCl, and stained with Alcian blue 8GS overnight before dye extraction with guanidine hydrochloride (200 μl, 6M). Absorbance of extracted dye was measured at 620 nm using a Multiskan Bichromatic 348 spectrophotometer and normalized to DNA content using SYBRgreen dye (excitation 485 nm and emission 535 nm) with a TECAN M200 spectrophotometer. These values were used to generate ECM accumulation concentrations and are expressed as percentage change from control micromasses. In some experiments, the FPR2/ALX receptor antagonist WRW_4_ (10 μM) was added to micromasses 10 minutes prior to 17*R*-RvD1 addition, and ECM accumulation was evaluated after 24 hours.

### Statistics.

Statistical analyses were performed with mean ± SEM, where *n* is the biological replicate (individual mice or human donors). Data were analyzed using either 2-tailed paired or unpaired Student’s *t* test, 1-way ANOVA with appropriate post hoc analysis, Mann-Whitney *t* test, or 2-way ANOVA with repeated measures where appropriate. Analyses were performed using GraphPad Prism; in all cases, *P* < 0.05 was considered significant. Multivariate analysis of arthritis metabololipidomics was performed using linear discriminant analysis, where bioactive lipid mediator predictors were entered simultaneously to the model (carried out in SPSS 23; IBM).

### Study approval.

Human peripheral blood was collected according to a protocol approved by Barts and the London Research Ethics Committee (London, United Kingdom [QMREC 2014:61]). Written informed consent was received from participants prior to inclusion in the study according to the declaration of Helsinki. All animal experiments were approved and performed under the guidelines of the Ethical Committee for the Use of Animals, Barts and The London School of Medicine, and in accordance with the UK Home Office regulations (Guidance on the Operation of Animals, Scientific Procedures Act, 1986).

## Author contributions

LVN designed and performed experiments, analyzed data, and wrote the manuscript. SEH performed experiments and analyzed data. JD and HHA analyzed metabololipidomics data. HRJ analyzed data and performed statistical analyses. OH assisted with flow cytometry analyses. DI provided unique tools for leukocyte chemotaxis. CNS and MP provided conceptual expertise and contributed to manuscript preparation.

## Supplementary Material

Supplemental data

## Figures and Tables

**Table 3:**
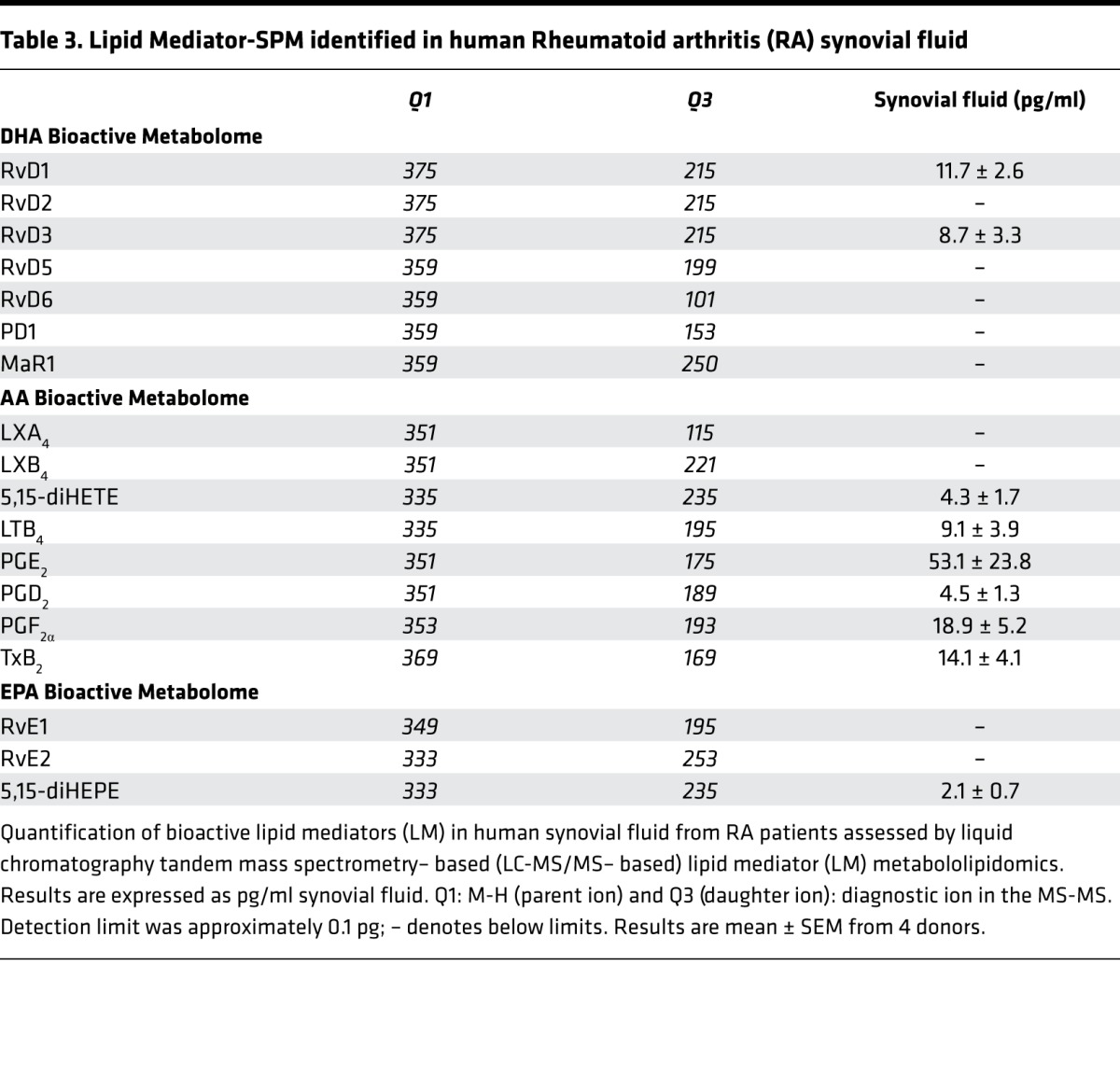
Lipid Mediator-SPM identified in human Rheumatoid arthritis (RA) synovial fluid

**Table 2:**
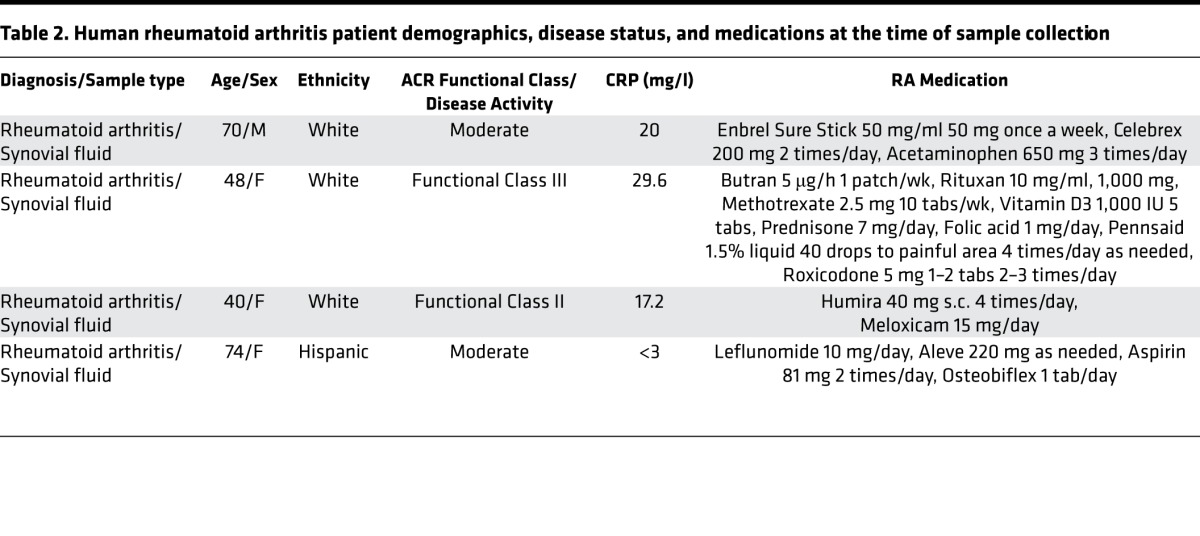
Human rheumatoid arthritis patient demographics, disease status, and medications at the time of sample collection

**Table 1:**
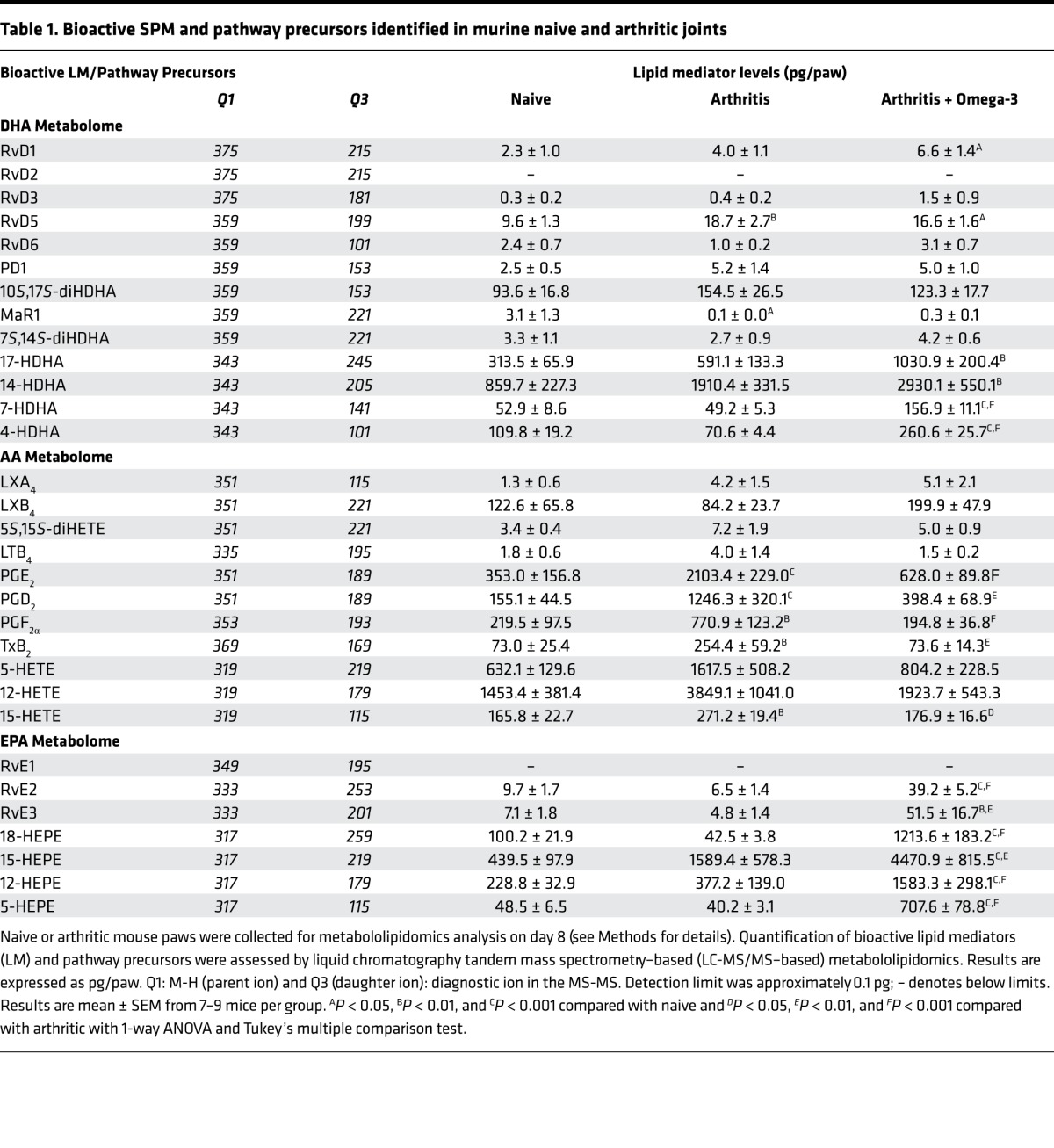
Bioactive SPM and pathway precursors identified in murine naive and arthritic joints
