# Proresolving and cartilage-protective actions of resolvin D1 in inflammatory arthritis

**DOI:** 10.1172/jci.insight.168728

**Published:** 2023-02-08

**Authors:** Lucy V. Norling, Sarah E. Headland, Jesmond Dalli, Hildur H. Arnardottir, Oliver Haworth, Hefin R. Jones, Daniel Irimia, Charles N. Serhan, Mauro Perretti

Original citation: *JCI Insight*. 2016;1(5):e85922. https://doi.org/10.1172/jci.insight.85922

Citation for this corrigendum: *JCI Insight*. 2023;8(3):e168728. https://doi.org/10.1172/jci.insight.168728

The authors recently became aware that representative illustrations presented in Figure 2A might be mistaken for original data. As this panel was used strictly for demonstrative purposes, it has been removed from the figure for clarity. The updated figure, figure legend, and description in the Results section are below.

The authors regret the errors.

*Identification of SPM in human RA synovial fluids*. We next profiled synovial fluid from human RA patients to determine the bioactive lipid mediator metabolome found within human joints (refer to Table 2 for patient demographics/treatment regimes). Using LC-MS/MS–based lipid mediator metabololipidomics, we identified mediators from all 3 major bioactive metabolomes in human synovial fluids including DHA-derived Rvs and AA-derived eicosanoids (Table 3). Similarly to murine arthritic joints, AA-derived autacoids classically associated with joint pain and inflammation were detected within the synovial effusate, including PGE_2_, PGD_2_, PGF_2α_, TXB_2_, and LTB_4_. In these fluids, we also identified the pathway marker for the protective lipoxin family 5,15-diHETE, as well as the D-series Rvs, RvD1, and RvD3. All of these mediators were identified in accordance with published criteria including matching RTs in LC and characteristic/diagnostic MS/MS fragmentation patterns (Figure 2). Of note, the levels of these proresolving mediators in these inflammatory exudates were detected within physiologically relevant concentrations (RvD1, ~31 pM, bioactive concentrations: 10 pM–100 nM; RvD3, ~23 pM, bioactive concentrations: 1 pM–10 nM) (refs. 8, 14, 15, and Table 3).

## Figures and Tables

**Figure 2:**
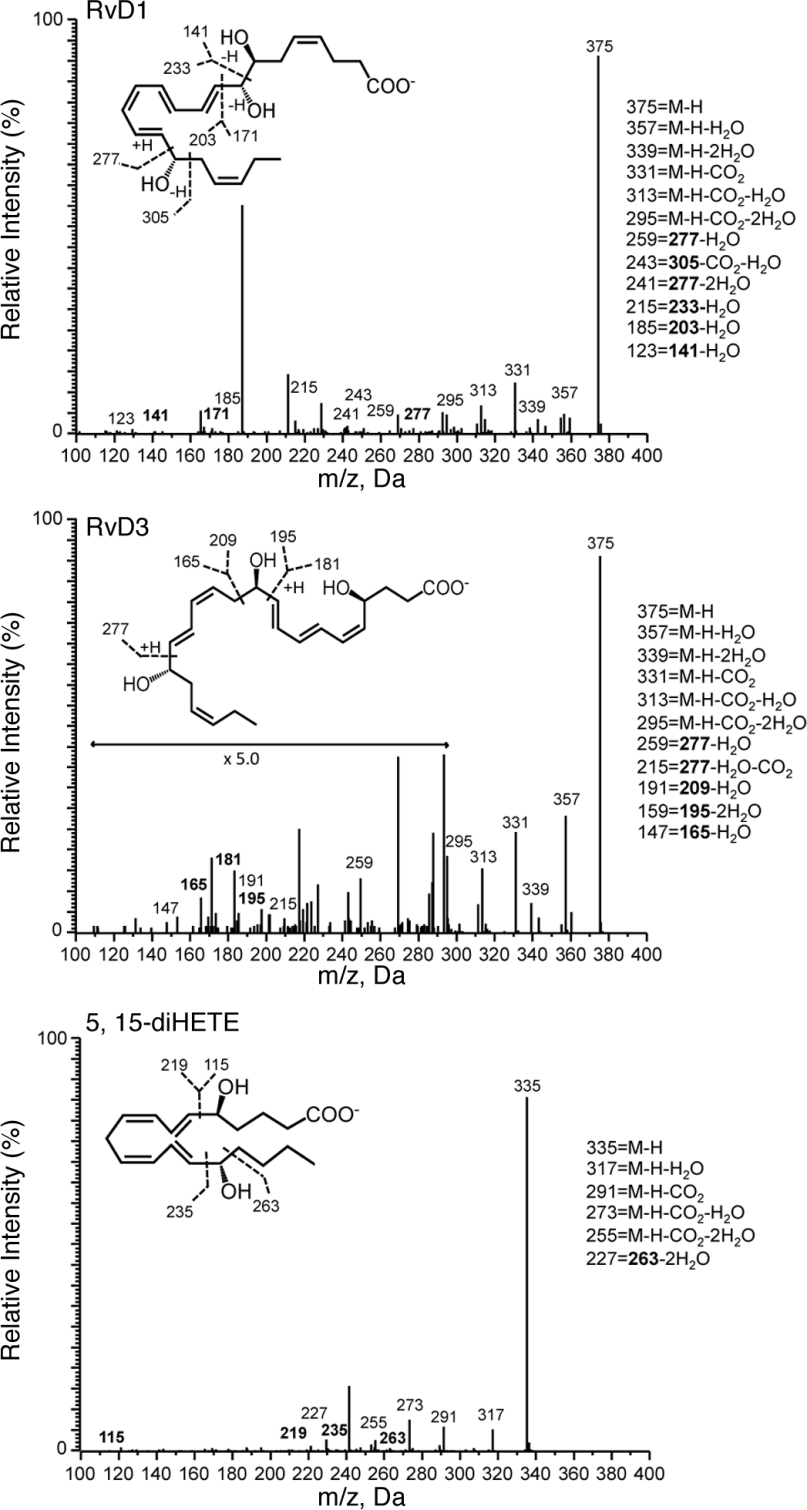
Identification of resolvin D1 (RvD1) and RvD3 in synovial fluids from rheumatoid arthritis (RA) patients. Lipid mediator levels were assessed following solid phase extraction by liquid chromatography tandem mass spectrometry–based (LC-MS/MS–based) metabololipidomics (see Methods for details). Accompanying MS/MS spectra utilized for identification. Refer to Table 2 for patient demographics and Table 3 for quantification of bioactive lipid mediators.

## Version 1 02/08/2023

Electronic publication

