# Supplementary material for: Proresolving and cartilage-protective actions of resolvin D1 in inflammatory arthritis
Source: JCI Insight. 2016 Apr 21;1(5):e85922. doi: 10.1172/jci.insight.85922 (PMC4855303; doi:10.1172/jci.insight.85922)
Supplement: Supplemental data [file jciinsight-1-85922-s001.pdf]

Supplemental Data

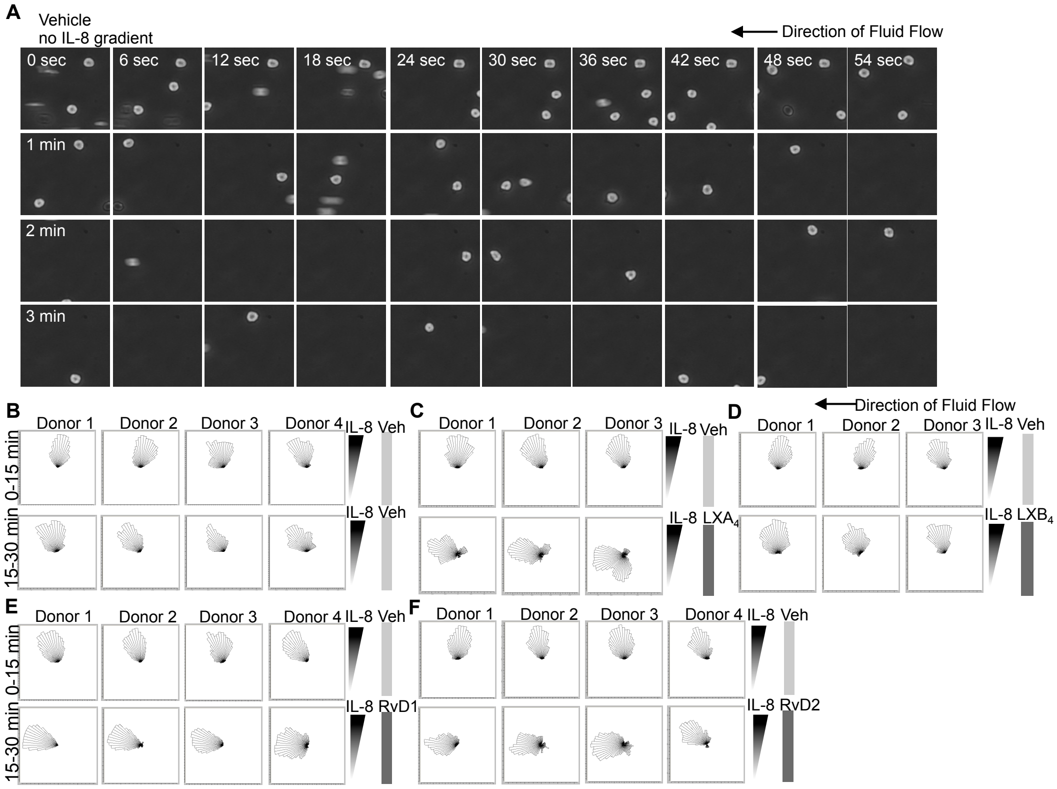

Supplemental Figure 1. Neutrophil migration trajectories following exposure to Specialised pro-resolving lipid mediators (SPM). Neutrophils were captured from whole blood of healthy volunteers on P-selectin and ICAM-1-coated microfluidics chambers. (A) Neutrophils remained rounded and rapidly detached in the absence of a chemotactic gradient. Cells were exposed to media plus vehicle (0.1% ethanol) and videos were captured in real-time over 15 min. Representative still images are shown for the first 4 min, taken every 6 sec. (B) Rose plots indicating migration trajectories from individual donors after exposure to an IL-8 gradient (10nM, 15min) followed by exposure to a set concentration of resolvin D1 (RvD1), RvD2, lipoxin A_4 (LXA_4) or LXB_4 (1nM, 15min) or vehicle together with the IL-8 gradient.

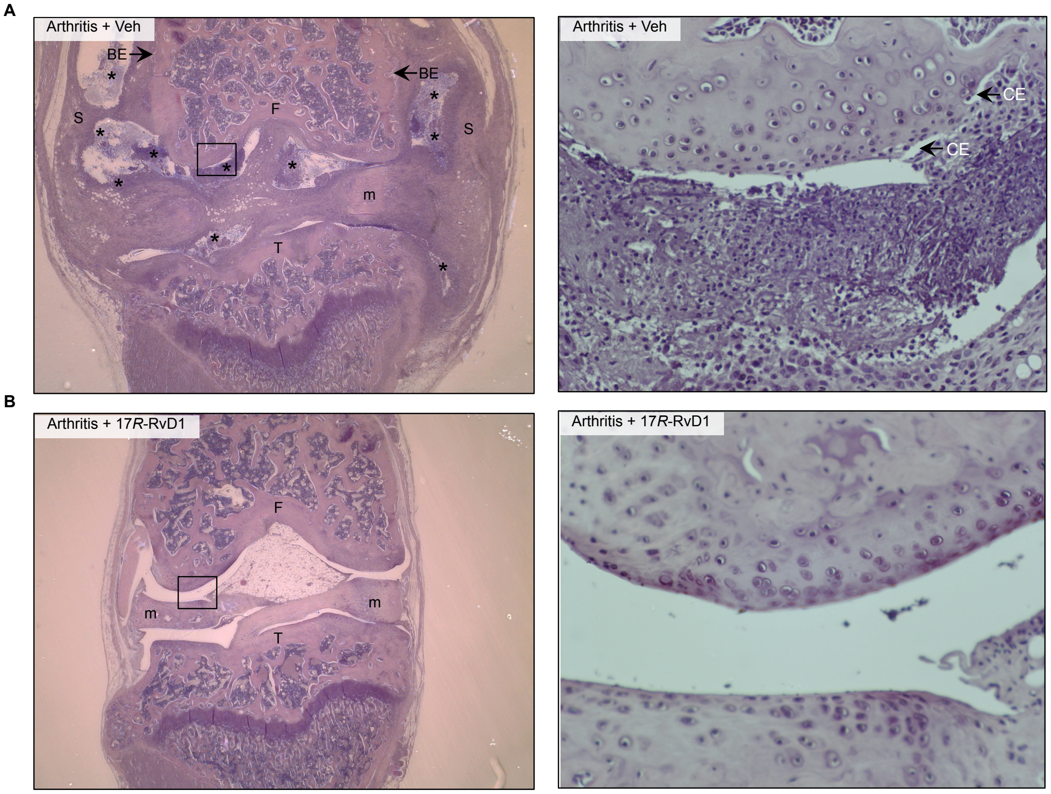

Supplemental Figure 2. Histopathology of murine knee joints is improved with 17*R*-RvD1 treatment. (A) Representative haematoxylin & eosin histology sections of knee joints from arthritic mice 8 days after arthritis induction following daily administration of vehicle (0.1% ethanol in PBS, i.p.) or (B) 17*R*-RvD1 (100ng, i.p.). Representative low (x4) and high (x20) power magnifications are shown for each genotype. F; femur, T; tibia, m; meniscus, PF; pannus formation, BE; bone erosion, S; synovitis, *; neutrophil infiltration, from *n*=5-7 mice per group.

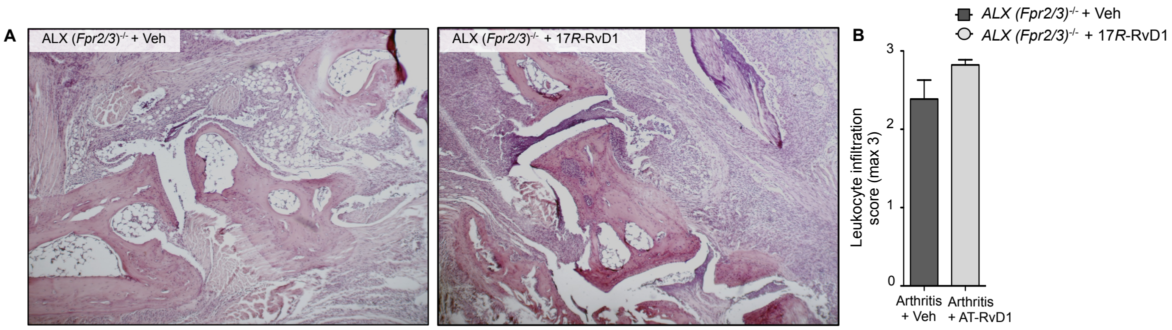

Supplemental Figure 3. Joint protection from 17*R*-RvD1 is lost in *Fpr2/3* (ALX) null mice. (A) Representative haematoxylin & eosin histology sections of murine arthritic hind paws 8 days after arthritis induction (x10 magnification). (B) Histological score calculated by degree of leukocyte infiltration (max. 3), *n*=5-6 mice per group.

	Relative Expression (AU)		
Gene	Arthritis	Arthritis + 17*R*-RvD1	% Change
Alox15	1.76 ± 0.73	3.74 ± 1.12	+ 112.5
Il-1β	22.56 ± 9.96	10.57 ± 2.58	- 53.2
Ly6g	4.85 ± 2.66	1.31 ± 0.35	- 73.0
Ptsg2	21.05 ± 10.78	6.35 ± 2.06	- 69.9

Supplemental Table 1. Comparative relative expression of inflammatory genes in murine arthritic joint tissue. Arthritic mouse paws were collected for gene analysis on day 8 (see methods for details). Relative expression values were calculated following normalization to endogenous housekeeping gene *Rpl32* and using the $2^{-(\Delta\Delta Ct)}$ method normalized to a naïve mouse (calibrator sample).
